# Modifying the Replication of Geminiviral Vectors Reduces Cell Death and Enhances Expression of Biopharmaceutical Proteins in *Nicotiana benthamiana* Leaves

**DOI:** 10.3389/fpls.2018.01974

**Published:** 2019-01-09

**Authors:** Andrew G. Diamos, Hugh S. Mason

**Affiliations:** Center for Immunotherapy, Vaccines, and Virotherapy, Biodesign Institute, School of Life Sciences, Arizona State University, Tempe, AZ, United States

**Keywords:** geminivirus replication, transient expression, Rep, bean yellow dwarf virus, 5′ UTR

## Abstract

Plants are a promising platform to produce biopharmaceutical proteins, however, the toxic nature of some proteins inhibits their accumulation. We previously created a replicating geminiviral expression system based on bean yellow dwarf virus (BeYDV) that enables very high-level production of recombinant proteins. To study the role of replication in this system, we generated vectors that allow separate and controlled expression of BeYDV Rep and RepA proteins. We show that the ratio of Rep and RepA strongly affects the efficiency of replication. Rep, RepA, and vector replication all elicit the plant hypersensitive response, resulting in cell death. We find that a modest reduction in expression of Rep and RepA reduces plant leaf cell death which, despite reducing the accumulation of viral replicons, increases target protein accumulation. A single nucleotide change in the 5′ untranslated region (UTR) reduced Rep/RepA expression, reduced cell death, and enhanced the production of monoclonal antibodies. We also find that replicating vectors achieve optimal expression with lower *Agrobacterium* concentrations than non-replicating vectors, further reducing cell death. Viral UTRs are also shown to contribute substantially to cell death, while a native plant-derived 5′ UTR does not.

## Introduction

Plant-based expression systems offer many potential advantages over traditional systems, including safety, speed, versatility, scalability, and cost (Gleba et al., 2014; Tusé et al., 2014; Chen and Davis, 2016; Nandi et al., 2016). The demonstration that plant-made pharmaceuticals can be glyco-engineered to have authentic human N-glycans, with greater homogeneity and subsequently greater efficacy than their mammalian-produced counterparts further underscores the potential of plant-based systems for the production of therapeutic proteins (Zeitlin et al., 2011; Hiatt et al., 2014; Strasser et al., 2014). However, high accumulation of foreign proteins, especially when ER-targeted, often puts significant stress on the plant cells. In some cases, this may lead to prohibitive levels of tissue necrosis that reduce yields (Hamorsky et al., 2015).

A plant-based transient expression system has been developed which uses the replication machinery from the geminivirus bean yellow dwarf virus (BeYDV) to substantially increase transgene copy number in the plant nucleus, with a subsequent increase in transcription of the target gene (Huang et al., 2009, 2010). This system has been used to produce high levels of vaccine antigens and pharmaceutical proteins in *Nicotiana benthamiana* leaves (Phoolcharoen et al., 2011; Lai et al., 2012; Moon et al., 2014; Kim et al., 2015; Diamos et al., 2016; Diamos and Mason, 2018). High levels of tissue necrosis have been noted when expressing certain proteins using BeYDV vectors, including Ebolavirus glycoprotein, hepatitis B core antigen, GII norovirus particles, monoclonal antibodies and other ER-targeted proteins (Phoolcharoen et al., 2011; Mathew et al., 2014, unpublished data). The factors contributing to cell death in the BeYDV system have not been thoroughly investigated.

The geminiviruses comprise a family of small (∼2.5 kb) single-stranded DNA viruses which replicate in the nucleus of host cells, associating with histones to form viral chromosomes (Pilartz and Jeske, 2003). BeYDV and other mastreviruses produce only four proteins: a coat protein and movement protein, which are produced by the virion sense DNA strand, and two replication proteins, Rep and RepA, produced on the complementary sense DNA strand (C1/C2 genes). Rep and RepA are produced from a single intron-containing transcript: RepA is the predominant protein product from the unspliced transcript, while a relatively uncommon excision of an intron alters the reading frame to produce Rep. Production of all viral proteins is driven by a single bidirectional promoter in the long intergenic region (LIR) which also contains the viral origin of replication. Both divergent transcripts converge at a short intergenic region (SIR), which has bidirectional transcription terminator signals and is suspected to be the origin of complementary strand synthesis (Liu et al., 1998).

Because geminiviruses produce few gene products, they are heavily reliant on host enzymes. The mastrevirus Rep protein, which is produced early in infection, is a multifunctional protein responsible for initiating rolling circle replication by nicking a conserved stem-loop sequence in the LIR. The majority of replication then occurs using cellular machinery to extend the free 3′ end of the nicked viral replicon, though it is likely that Rep recruits many of the involved cellular factors (Gutierrez, 1999). Rep also plays a role in ligating newly synthesized DNA to create circular viral genomes and possesses helicase activity (Choudhury et al., 2006). In the bipartite begomoviruses, Rep has been shown to form homo-oligomers, or possibly hetero-oligomers with RepA or other proteins, which may play a role in replication (Horváth et al., 1998; Krenz et al., 2011).

A primary function of RepA is thought to be the creation of a cellular environment suitable for replication. Some evidence suggests this occurs by binding retinoblastoma-related proteins, which are involved in cell cycle regulation. With RepA bound, previously sequestered transcription factors are able to initiate S-phase gene expression, creating the cellular machinery necessary for viral replication (Gutierrez et al., 2004). An LxCxE motif has been shown to contribute to retinoblastoma-related protein binding (Ruschhaupt et al., 2013). However, other functions of RepA, many of which are still unidentified, have also been shown to enhance viral replication. A set of proteins known as GRAB proteins, which are involved in leaf development and senescence, have also been found to interact with RepA (Lozano-Durán et al., 2011).

Viral proteins are often potent inducers of the plant hypersensitive response, an immune defense mechanism that triggers the release of reactive oxygen species, autophagy, host translation shutoff, and programmed cell death in response to pathogen infection (Dodds and Rathjen, 2010; Zhou et al., 2014; Zorzatto et al., 2015). In the begomoviruses, the bean dwarf mosaic virus nuclear shuttle protein (NSP) was shown to activate the hypersensitive response in bean plants (Garrido-Ramirez et al., 2000), and this activity was mapped to the N-terminus of the NSP (Zhou et al., 2007). As a countermeasure, the TrAP protein from tomato leaf curl New Delhi virus prevents the activation of the hypersensitive response generated by its NSP (Hussain et al., 2007). Additionally, the NSP is known to interact with a host immune NB-LRR receptor-like kinase to enhance virus pathogenicity, and is involved in preventing translation shutoff in response to virus infection (Sakamoto et al., 2012; Zhou et al., 2014). The Rep protein from African cassava mosaic virus also elicited the hypersensitive response in *N. benthamiana* (van Wezel et al., 2002), and it was further reported that altering a single amino acid reversed hypersensitive response induction without affecting protein function (Jin et al., 2008). While many studies have focused on the begomoviruses, the role of the hypersensitive response during mastrevirus infection has not been investigated.

In this study, we have created a system that allows separate and controlled expression of BeYDV Rep and RepA. Using this system, we investigate how Rep and RepA control replication, and contribute to leaf cell death. By reducing expression of Rep and RepA, BeYDV-based expression vectors elicit lower levels of cell death, with a corresponding increase in the production of vaccine antigens and monoclonal antibodies. We also explore other factors contributing to cell death in plant expression systems, including the relationship between vector replication and *Agrobacterium* concentration, and the contribution of viral elements to cell death.

## Materials and Methods

### Vector Construction

A series of expression vectors containing promoters of varying strengths were created to express rep Rep and RepA. The Ubi3 promoter was obtained from pUbi3-GUS (Garbarino and Belknap, 1994) by BseRI (T4 blunt) PstI digestion, and ligated into pRep110 (Huang et al., 2009) digested SbfI (T4 blunt) and XhoI, to create pRep107. The Ubi3 promoter with ubiquitin fusion was excised from pUbi3-GUS by PstI-NcoI digestion, and ligated into pRep110 digested SbfI-SacI along with C1/C2 excised from pBY036 digested NcoI-SacI to create pRep106. The soybean vspB promoter was obtained from pGUS220 (Mason et al., 1993) by HindIII-NcoI digestion and ligated with pRep110 digested HindIII-SacI and pBY034 digested NcoI-SacI to create pRep108. The *Agrobacterium* nopaline synthase (NOS) promoter was obtained from pGPTV-Kan (Becker et al., 1992) by HindIII-NcoI digestion and ligated into pBI101 (Jefferson et al., 1987) along with C1/C2 excised from pBY036 digested NcoI-SacI to create pRep111.

The intron-deleted form of BeYDV rep was previously described (Mor et al., 2003). For RepA vectors, the sequence following the RepA stop codon was deleted and an additional stop codon was inserted in the Rep reading frame to prevent further translation. To accomplish this, a primer RepA-Sac-R (5′-CGGAGCTCTATGTTAATTGCTTCCACAATGGGAC-3′) designed to insert a stop codon and create a SacI site at the end of the RepA coding sequence was used to amplify RepA from pRep110 along with primer TEV (5′-GCATTCTACTTCTATTGCAGC-3′). The product was digested ClaI-SacI, and ligated into pRep110 digested likewise to yield pRepA110. XhoI-SacI or NcoI-SacI fragments containing either the deleted intron form of Rep excised from pBY037, or RepA excised from pRepA110, were ligated into expression vectors containing the promoters Ubi (pRep106), UbiF (pRep107), VspB (pRep108), or NOS (pRep111) to generate Rep and RepA expressing vectors.

To create BeYDV expression vectors that required Rep/RepA to be supplied in trans, Rep/RepA were deleted from the Norwalk virus capsid protein (NVCP)-expressing vector pBYR2e-sNV or the rituximab-expressing vector pBYR2e-MRtx (Diamos et al., 2016) by BamHI digestion and self-ligation of the backbone vector to yield pBY-2e-sNV and, pBY-2e-MRtx respectively. The empty replicon vector pBY-EMPTY was created by excising the PstI-SacI fragment from pKS-RT38, which contains the potato pinII terminator region derived from pRT38 (Thornburg et al., 1987), and ligating it into pBY-GFP (Huang et al., 2009) digested SbfI-SacI. To introduce a **A**ACATG to **C**ACATG mutation to the 5′ UTR of Rep/RepA, the primer LIRc-Nhe2-R (5′-taGCTAGCAGAAGGCATGTGGTTGTGACTCCGAGGGGTTG-3′) containing the mutation was used to amplify the modified LIR from pBY027 with primer M13F. The polymerase chain reaction (PCR) product was digested NheI-AgeI and ligated into pBYR2e-GFP digested BspDI-AgeI along with the rep-containing NheI-BspDI fragment from pBYR2e-GFP to create pBY-R2-GFP. Vectors containing NbPsaK, PEMV and BYDV 3′ and 5′ UTRs were previously described (Diamos et al., 2016; Diamos and Mason, 2018). Further details are available upon request.

### Agroinfiltration of *Nicotiana benthamiana* Leaves

Binary vectors were separately introduced into *Agrobacterium tumefaciens* GV3101 or EHA105 by electroporation. The resulting strains were verified by restriction digestion or PCR, grown overnight at 30°C, and used to infiltrate leaves of 5- to 6-week-old *N. benthamiana* maintained at 23–25°C. Briefly, the bacteria were pelleted by centrifugation for 5 min at 5,000 *g* and then resuspended in infiltration buffer (10 mM 2-(*N*-morpholino)ethanesulfonic acid (MES), pH 5.5 and 10 mM MgSO_4_) to OD_600_ = 0.2, unless otherwise described. When mixing two constructs, each *Agrobacterium* concentration was instead set to OD_600_ = 0.4, and then mixed 1:1. Similarly, for three constructs, each was set to OD_600_ = 0.6, and mixed 1:1:1. The resulting bacterial suspensions were injected by using a syringe without needle into fully expanded leaves (9–12 cm long) through a small puncture (Huang et al., 2006). Plant tissue was harvested after 5 DPI, or as stated for each experiment. Leaves producing GFP were photographed under UV illumination generated by a B-100AP lamp (UVP, Upland, CA, United States).

### Protein Extraction

Total protein extract was obtained by homogenizing agroinfiltrated leaf samples with 1:5 (*w:v*) ice cold extraction buffer (25 mM sodium phosphate, pH 7.4, 100 mM NaCl, 1 mM EDTA, 0.1% Triton X-100, 10 mg/mL sodium ascorbate, 0.3 mg/mL phenylmethylsulfonyl fluoride) using a Bullet Blender machine (Next Advance, Averill Park, NY, United States) following the manufacturer’s instruction. To enhance solubility, homogenized tissue was rotated at room temperature or 4°C for 30 min. The crude plant extract was clarified by centrifugation at 13,000 *g* for 10 min at 4°C. Necrotic leaf tissue has reduced water weight, which can lead to inaccurate measurements based on leaf mass. Therefore, extracts were normalized based on total protein content by Bradford protein assay kit (Bio-Rad, Hercules, CA, United States) with bovine serum albumin as standard.

### SDS-PAGE and Western Blot

Clarified plant protein extract was mixed with sample buffer (50 mM Tris-HCl, pH 6.8, 2% SDS, 10% glycerol, 0.02% bromophenol blue) and separated on 4–15% polyacrylamide gels (Bio-Rad, Hercules, CA, United States). For reducing conditions, 0.5 M dithiothreitol was added, and the samples were boiled for 10 min prior to loading. Polyacrylamide gels were either transferred to a PVDF membrane or stained with Coomassie stain (Bio-Rad, Hercules, CA, United States) following the manufacturer’s instructions. For Rep/RepA detection, the protein transferred membranes were blocked with 5% dry milk in PBST (PBS with 0.05% Tween-20) for 1 h at 37°C and probed in succession with rabbit anti-Rep (antibodies raised against an N-terminal 154 amino acid fragment of Rep/RepA) diluted 1:2000 and goat anti-rabbit IgG-horseradish peroxidase conjugated (Sigma-Aldrich, St. Louis, MO, United States) diluted 1:10,000 in 1% PBSTM. Bound antibody was detected with ECL reagent (Amersham, Little Chalfont, United Kingdom). For GFP detection, the 26 kDa fluorescent GFP band was quantified by gel densitometry using ImageJ software.

### Protein Quantification by ELISA

GI and GII norovirus capsid concentration was analyzed by sandwich ELISA. A rabbit polyclonal anti-GI or anti-GII antibody was bound to 96-well high-binding polystyrene plates (Corning, Corning, NY, United States), and the plates were blocked with 5% non-fat dry milk in PBST. After washing the wells with PBST (PBS with 0.05% Tween 20), the plant extracts were added and incubated. The bound norovirus capsids were detected by incubation with guinea pig polyclonal anti-GI or anti-GII antibody followed by goat anti-guinea pig IgG-horseradish peroxidase conjugate. The plate was developed with TMB substrate (Thermo Fisher Scientific, Waltham, MA, United States) and the absorbance was read at 450 nm. Plant-produced GI or GII capsids were used as the reference standard (Kentucky Bio Processing, Owensboro, KY, United States).

For rituximab quantification, plant protein extracts were analyzed by ELISA designed to detect the assembled form of mAb (with both light and heavy chains) as described previously (Giritch et al., 2006). Briefly, plates were coated with a goat anti-human IgG specific to gamma heavy chain (Southern Biotech, Birmingham, AL, United States). After incubation with plant protein extract, the plate was blocked with 5% non-fat dry milk in PBST, then incubated with a HRP-conjugated anti-human-kappa chain.

### Plant DNA Extraction and Replicon Quantification

Total DNA was extracted from 0.1 g plant leaf samples using the DNeasy Plant Mini Kit (Qiagen) according to the manufacturer’s instructions. DNA (∼1 μg) was separated on 1% agarose gels stained with ethidium bromide. The replicon DNA band intensity was quantified using ImageJ software, using the high molecular weight plant chromosomal DNA band as an internal loading control. Columns represent means ± standard deviation from three or more independently infiltrated samples.

### Reverse Transcription-Polymerase Chain Reaction

Total RNA was extracted from 0.1 g leaf samples using the RNeasy Plant Mini Kit (Qiagen) according to the manufacturer’s instructions. Residual DNA was removed using the DNA-Free system (Ambion). First-strand cDNA was synthesized from 1 μg of total RNA primer using the Superscript III First Strand Synthesis System (Invitrogen) according to the manufacturer’s instructions using oligo dT_22_ primer. Reverse transcription-polymerase chain reaction (RT-PCR) was performed using primers RepF (5′-ACCCCAAGTGCTCATCTC-3′) and RepR1 (5′-GCGACACGTACTGCTCA-3′) to detect Rep and RepA transcripts.

## Results

### Controlled Production of Rep and RepA in Plant Leaves

**FIGURE 1:**
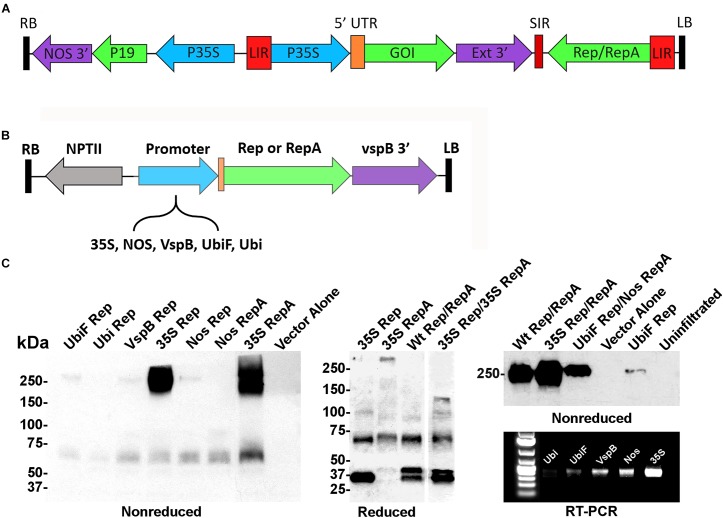
Controlled expression of Rep and RepA in *Nicotiana benthamiana* leaves. **(A)** Generalized schematic representation of the BeYDV vectors used in this study. RB and LB, the right and left borders of the T-DNA region from *Agrobacterium*; NOS 3′, the nopaline synthase terminator from *Agrobacterium*; P19, the RNA silencing suppressor from tomato bushy stunt virus; 35S, the 35S promoter from cauliflower mosaic virus; LIR, the long intergenic region from BeYDV; 5′ UTR, the 5′ untranslated region as described in each experiment; GOI, the gene of interest, as described in each experiment; Ext 3′, the 3′ region from the tobacco extensin gene; SIR, the short intergenic region from BeYDV; Rep/RepA, the replication proteins from BeYDV, which are either present in wild-type form, or are deleted or mutated as described in each experiment. **(B)** Generalized schematic representation of the T-DNA region of the separated Rep/RepA vectors used in this study. NPTII, kanamycin resistance cassette; VspB 3′, vegetative storage protein B gene terminator from soybean; Promoter, various promoters as described with 5′ UTR from tobacco etch virus; NOS, the nopaline synthase promoter from *Agrobacterium*; VspB, the vegetative storage protein B promoter from soybean; Ubi, the ubiquitin-3 promoter from potato; UbiF, Ubi with ubiquitin fusion **(C)** *Agrobacterium* carrying the indicated T-DNA vectors mixed to a final OD of 0.2 for each construct and were infiltrated into the leaves of *N. benthamiana*. After 4 days post-infiltration (DPI), leaf tissue samples were harvested, and protein extracts were analyzed by reducing or non-reducing western blot. In the “Reduced” gel, the lane “35S Rep/35S RepA” was pasted from a different gel than the other lanes (two representative gels of Rep/RepA expression shown in Supplementary Figures S1, S2 were combined into a single panel). For RT-PCR, RNA was extracted from leaf samples and 50 ng of converted cDNA were PCR amplified with Rep-specific primers.

In the BeYDV expression system (Figure 1A), production of Rep/RepA leads to excision, circularization, and replication of any gene expression cassette flanked by the *cis-*acting LIRs. Previously, we showed that a Rep/RepA-supplying vector could be delivered in trans to amplify a replication-deficient BeYDV containing the LIRs but lacking Rep/RepA (Huang et al., 2009). However, this system was only capable of producing Rep and RepA together, at constant high levels under the control of the strong 35S promoter from cauliflower mosaic virus. To create a modular system to study vector replication, a series of *Agrobacterium* T-DNA expression vectors were constructed that separately expressed either Rep or RepA under the control of five different promoters: the 35S promoter, the nopaline synthase promoter from *Agrobacterium* (NOS), the vegetative storage protein B promoter from soybean (vspB), or the ubiquitin-3 promoter from potato with (UbiF) or without (Ubi) ubiquitin fusion (Figure 1B). To characterize the expression of Rep and RepA by these vectors, they were infiltrated into the leaves of *N. benthamiana* and analyzed by western blot and RT-PCR. Rep and RepA from the related wheat dwarf virus are known to form oligomeric complexes (Missich et al., 2000). Antibodies targeting both Rep and RepA produced together in their native wild-type configuration reacted strongly with non-reduced protein extracts, revealing large complexes near 250 kDa in size. RepA produced two distinct high molecular weight bands, whereas Rep produced only a single resolvable band (Figure 1C, non-reduced). However, when Rep and RepA were expressed together, only a single band at the size of rep alone was observed (Figure 1C, right panel). Under reducing conditions, Rep (predicted 39 kDa) produced predominately monomeric 35–40 kDa bands, while RepA (predicted 33 kDa) showed 65–75 kDa bands suggestive of oligomeric forms. Interestingly, when both Rep and RepA were coexpressed, a slightly larger 45–50 kDa band of unknown origin also appeared (Figure 1C). RT-PCR and western analysis both showed that the 35S construct far exceeded the other expression vectors, followed by the NOS, vspB, UbiF constructs, with the unfused Ubi construct providing the weakest expression (Figure 1C).

### The Ratio of Rep and RepA Is Important for Efficient BeYDV Replication

**FIGURE 2:**
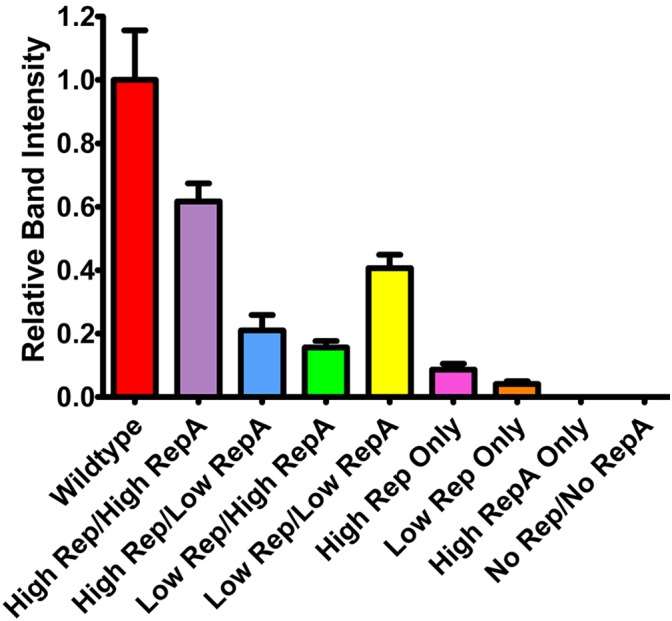
Replicon accumulation by differential Rep/RepA expression. Leaves of *N. benthamiana* were agroinfiltrated with either low (UbiF) or high (35S) expression vectors producing combinations of Rep and/or RepA, along with the replicon vector pBY-2e-NVCP. Leaf tissue samples were harvested at 4 DPI, and 1 μg of extracted total DNA was separated and visualized by ethidium bromide stained agarose gel electrophoresis. The relative intensity of replicon bands was quantified with ImageJ software. Error bars are means ± standard deviation of three or more independently infiltrated samples.

To determine the effects of altered Rep and RepA expression on replicon amplification, a replicon vector pBY-2e-sNV encoding a synthetic GI norovirus capsid protein (NVCP) was coinfiltrated with Rep and RepA supplying vectors. For simplicity, further experiments were performed with either UbiF vectors for low expression or 35S vectors for high expression, as no major notable differences were observed among the lower expressing constructs. The vector pBYR2e-sNV, which contains the wild-type Rep/RepA configuration driven by the native LIR promoter, was used as a control. In agreement with previous data on mastrevirus replication (Huang et al., 2009; Ruschhaupt et al., 2013), no replication was detected when RepA alone was supplied, and very low replication was detected when Rep was supplied alone with either a weak or strong promoter (Figure 2). However, coinfiltration of both Rep and RepA resulted in robust replication (Figure 2). Interestingly, overproduction of either Rep or RepA relative to the other resulted in impaired replication, suggesting that the relative abundance of each protein is important for efficient replication (Figure 2). Although expression of Rep and RepA by the strong 35S promoter was comparable to or exceeded wild-type expression levels (Figure 1C), the wild-type configuration resulted in a consistent increase in replicon accumulation, possibly due to differing to ratios of Rep/RepA expression (Figure 2). These results show that the level of vector replication can be controlled by differential expression of Rep and RepA.

### Reducing Vector Replication Reduces Cell Death and Increases Transgene Expression

**FIGURE 3:**
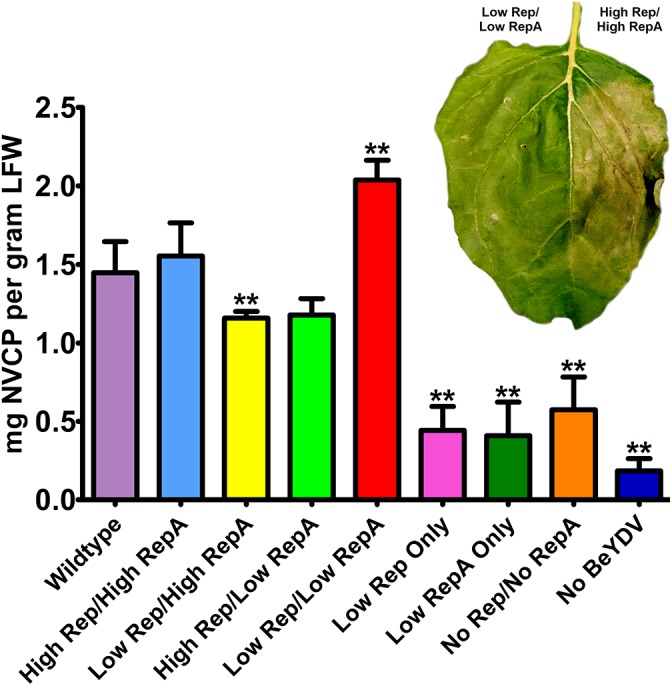
Norwalk virus capsid protein (NVCP) production by differential Rep/RepA expression. Leaves were agroinfiltrated with either low (UbiF) or high (35S) expression vectors producing combinations of Rep and/or RepA, along with the replicon vector pBY-2e-NVCP. Leaf tissue samples were harvested at 4–5 DPI, and protein extracts were analyzed for NVCP production by ELISA. Bars represent means ± standard deviation from three or more independently infiltrated leaf samples. (^∗∗^) Indicates *p* < 0.05 by Student’s *t*-test compared to wild-type Rep/RepA. Representative leaves were imaged at 4–5 DPI under visible light to monitor the development of necrosis.

Previously, we have shown that coinfiltration of a replicon vector and a Rep/RepA-supplying vector encoding both Rep and RepA together in the native configuration enhances the production of target proteins (Mor et al., 2003; Huang et al., 2009). To further characterize the relationship between replicon amplification and target protein accumulation, the production of NVCP from replicons amplified with variable levels of Rep and RepA was measured by ELISA. The control vector psNV120e contains no BeYDV elements and thus cannot replicate, whereas pBY-2e-sNV contains the intergenic regions from BeYDV necessary for replication. Interestingly, even in the absence of Rep and RepA, pBY-2e-sNV substantially increased NVCP expression by 3.1-fold compared to psNV120e, accumulating NVCP at 0.57 mg/g LFW (Figure 3A). NVCP expression was further enhanced by an additional 2.7-fold when pBY-2e-sNV was coinfiltrated with 35S-driven Rep/RepA or when Rep/RepA were supplied by the wild-type LIR promoter, yielding NVCP at approximately 1.5 mg/g LFW (Figure 3A). Unexpectedly, coinfiltration with vectors supplying Rep and RepA at lower than wild-type levels produced the highest yield of NVCP, reaching 2.0 mg/g LFW. The increase in NVCP expression was notably associated with a reduction in plant cell death (Figure 3B). Among replicating vectors, NVCP expression was lowest when the production of either Rep or RepA was substantially higher relative to the other, consistent with our data showing that these combinations have impaired replication (Figure 3B).

### Rep and RepA Induce Leaf Cell Death

**FIGURE 4:**
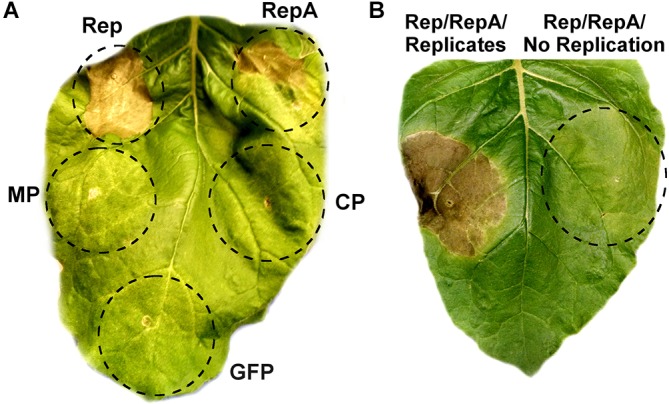
Rep/RepA expression induces chlorosis and cell death. **(A)** Leaves were agroinfiltrated with vectors supplying high levels of Rep, RepA, GFP, or an empty vector with coding sequences removed. Leaves were monitored for tissue necrosis, and representative images were taken at 8 DPI. **(B)** Leaves were agroinfiltrated with either Rep/RepA (pRep110) alone, or both pRep110 and the empty replicon vector pBY-EMPTY. Image was taken at 8 DPI.

As viral proteins are often contributors to cell death, the individual contribution of BeYDV proteins to plant leaf necrosis was investigated. Vectors using the strong 35S promoter to express either rep, RepA, the movement and coat proteins from BeYDV, or GFP were individually agroinfiltrated into *N. benthamiana* leaves and monitored for leaf tissue health. Both Rep and RepA produced chlorotic leaf tissue by 3–5 DPI which developed signs of leaf browning and eventually progressed to necrotic lesions by 6–10 DPI, whereas the movement protein, coat protein, and GFP did not produce any notable symptoms (Figure 4A). The progression of leaf necrosis was greater for Rep than RepA, and the development of necrosis was quicker in older leaves than in younger leaves (data not shown). We also investigated whether replicon amplification itself might contribute to leaf necrosis. The vector pRep110, which expresses Rep/RepA together in the native configuration and is insufficient to cause significant cell death on its own, was coinfiltrated with either pBY-EMPTY, which contains the *cis*-elements necessary for replication but with gene coding sequences replaced with a terminator, or pPS1, which contains no replication elements. Leaf spots infiltrated with pBY-EMPTY and pRep110 produced chlorotic leaf tissue after 3–4 DPI, and necrotic leaf tissue after 6–8 DPI, whereas leaf spots infiltrated with pPS1 and Rep/RepA did not produce necrotic tissue up to 10 DPI (Figure 4B).

### Expression of Toxic Proteins Is Enhanced by Reducing Rep/RepA Expression

**FIGURE 5:**
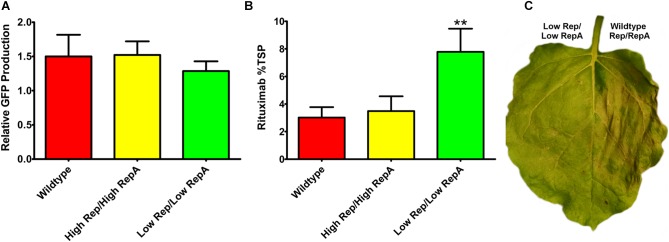
Expression of GFP and rituximab with modified Rep/RepA vectors. Leaves were coinfiltrated with modified Rep/RepA vectors and replicon vectors expressing either **(A)** GFP or **(B)** rituximab. For GFP analysis, protein extracts were separated on SDS-PAGE gels, and the GFP band intensity was quantified using ImageJ software. Columns are means ± standard deviation of three or more independently infiltrated samples. For rituximab, antibody production was quantified by IgG ELISA. Total soluble protein was determined by Bradford assay using bovine serum albumin and standard. Columns represent means ± standard deviation from three or more independently infiltrated leaf samples. (^∗∗^) Indicates *p* < 0.05 by Student’s *t*-test compared to wild-type Rep/RepA. **(C)** Representative leaves were imaged at 4–5 DPI under visible light to monitor the development of necrosis.

To determine whether a modest reduction in Rep/RepA would also benefit the expression of other transgenes, reduced Rep/RepA vectors were coinfiltrated with either pBY-2e-GFP, encoding GFP, or with pBY-2e-MRtx encoding the heavy and light chains of the monoclonal antibody rituximab. These vectors were compared to replicating vectors containing Rep/RepA in the wild-type configuration driven by the native LIR promoter: pBYR2e-GFP and pBYR2e-MRtx. We have previously shown pBYR2e-GFP accumulates high levels of GFP (Diamos et al., 2016). While GFP is known to be well-tolerated even when produced at very high levels in *N. benthamiana* leaves, the monoclonal antibody rituximab was found to induce a strong cell death response with BeYDV vectors (Diamos et al., 2016). A small but statistically insignificant decrease was observed in GFP expression when low Rep/RepA were supplied, compared to high Rep/RepA or wild-type, and no cell death was observed with any vector (Figure 5A, and data not shown). By contrast, heavy cell death was observed when rituximab was expressed with wild-type or high Rep/RepA, but not when Rep/RepA were reduced, and this reduction in cell death was correlated with a notable ∼2-fold increase in antibody accumulation (Figures 5B,C). These results suggest that reducing Rep/RepA from the wild-type level enhances the production of otherwise toxic proteins.

### A Single Nucleotide Mutation Reduces Replication, Reduces Cell Death, and Increases Antibody Production

**FIGURE 6:**
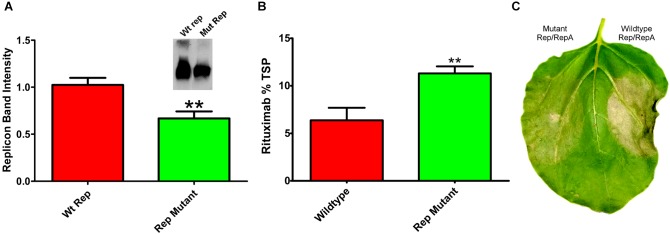
Characterization of Rep/RepA 5′ UTR mutant. Leaves of *N. benthamiana* were agroinfiltrated with the rituximab-producing replicon vector with (pBYe-R2-MRtx) or without (pBYR2e-MRtx) a mutated Rep/RepA 5′ UTR and analyzed after 4–5 DPI for **(A)** replicon band intensity quantified from 500 ng total DNA by ethidium bromide stained agarose gel or (inset) western blot. **(B)** Rituximab production by IgG ELISA, and **(C)** leaf necrosis photographed at 5 DPI. (^∗∗^) Indicates *p* < 0.05.

Reducing replication by supplying Rep and RepA on separate vectors requires simultaneous delivery of three different *Agrobacterium* cultures. To construct a simplified vector with reduced expression of Rep and RepA, single nucleotide mutations were created in the native 5′ UTR of Rep/RepA at the -3 position from the Rep/RepA start codon. These mutations were designed to provide a less favorable sequence context for translation initiation, which has been shown to favor A or G in the -3 position for dicot plants (Sugio et al., 2010). We found that a **A**ACATG to **C**ACATG mutation (where ATG indicates the rep start codon) reduced both Rep/RepA accumulation and replicon amplification (Figure 6A) by approximately 40%, similar to the results observed with low-expressing separated Rep/RepA vectors. To characterize expression and cell death with this vector, rituximab was produced with or without the mutation. As expected, the Rep/RepA mutant had reduced cell death (Figure 6C) and increased antibody production, reaching 10% TSP or approximately 0.8–1.0 g rituximab per kg leaf tissue (Figure 6B). These results indicate that vector replication can be reduced with a single change from the wild-type Rep/RepA gene.

### Replicating Vectors Require Lower *Agrobacterium* Concentrations Than Non-replicating Vectors

**FIGURE 7:**
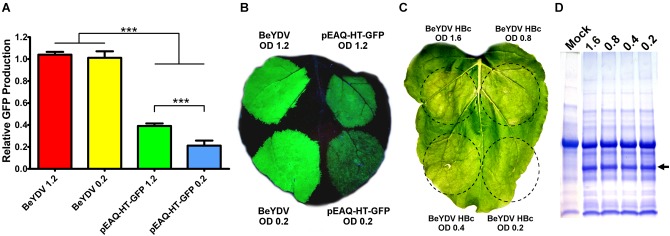
Replicating vectors require lower *Agrobacterium* concentration for optimal expression. Leaves of *N. benthamiana* were agroinfiltrated with the GFP-expressing BeYDV vectors or the non-replicating vector pEAQ-HT-GFP at the indicated OD_600_ values. **(A)** Leaf spots were assayed for GFP production by SDS-PAGE followed by quantification of fluorescence band intensity by ImageJ software. **(B)** Leaf images under UV light or **(C)** visible light. **(D)** Protein extractions from leaf spots agroinfiltrated at the indicated OD_600_ values with a BeYDV vector expressing an HBc heterodimer were visualized by SDS-PAGE with Coomassie staining. Arrow indicates HBc heterodimer band. A representative mock-infiltrated protein extract from a different gel is shown at left for comparison. (^∗∗∗^) Indicates *p* < 0.01.

In *N. benthamiana* leaves, we have previously found that *Agrobacterium* strain EHA105 reduces leaf necrosis relative to other commonly used *Agrobacterium* strains when used to deliver replicating BeYDV vectors (Diamos et al., 2016). Many non-replicating vector systems use high *Agrobacterium* concentrations of around an OD_600_ of 1.2 (Sainsbury et al., 2009). To investigate the relationship between *Agrobacterium* concentration and vector replication, a replicating BeYDV vector expressing GFP was infiltrated at various *Agrobacterium* concentrations. No significant differences in GFP expression were observed until the OD_600_ was reduced below 0.2 (Figure 7A). By contrast, GFP expression with pEAQ-HT-GFP (Sainsbury et al., 2009) was reduced by nearly half when the *Agrobacterium* OD_600_ was decreased from 1.2 to 0.2 (Figure 7B). Similar results were found with other non-replicating vectors (data not shown). While GFP was well-tolerated at all *Agrobacterium* concentrations tested, we reasoned that the added *Agrobacterium* load may be less tolerable with more toxic proteins. To further evaluate the relationship between *Agrobacterium* concentration and cell death, replicating BeYDV vectors expressing hepatitis B core antigen tandem-linked heterodimers (Peyret et al., 2015) were infiltrated at decreasing *Agrobacterium* concentrations. *Agrobacterium* OD_600_ concentrations of 1.6 and 0.8 produced visible leaf necrosis, while 0.4 and 0.2 did not (Figure 7C). No differences in hepatitis B core antigen expression were observed (Figure 7D). Taken together, these data show that replicating BeYDV vectors provide optimal expression with lower *Agrobacterium* concentrations than non-replicating vectors, allowing further reductions in cell death.

### Viral Flanking Regions Contribute to Cell Death

**FIGURE 8:**
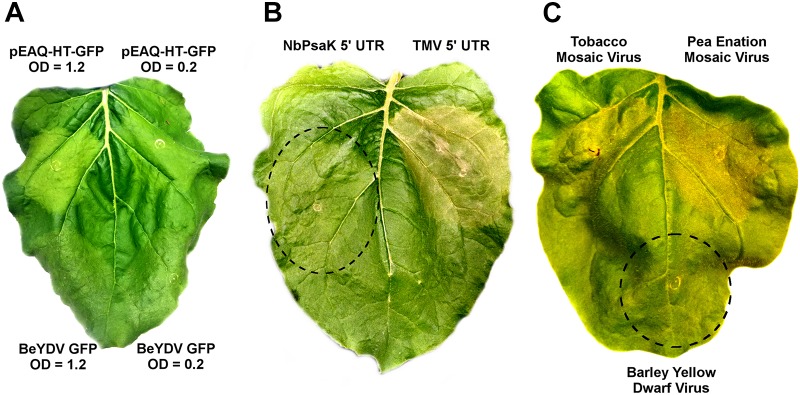
Virus-derived 5′ and 3′ untranslated regions induce cell death. **(A)** Leaves of *N. benthamiana* were agroinfiltrated with pEAQ-HT-GFP, which contains the CPMV 5′ and 3′ UTRs, or the BeYDV GFP vector pBYR2eK2Mc-GFP, at the indicated OD_600_ values and imaged under visible light at 5 DPI. **(B)** Leaves were agroinfiltrated with a BeYDV rituximab vectors containing either the NbPsaK 5′ UTR or TMV 5′ UTR and imaged at 5 DPI. **(C)** BeYDV GFP vectors containing the 5′ and 3′ UTRs from tobacco mosaic virus, pea enation mosaic virus, and barley yellow dwarf virus were agroinfiltrated and imaged under visible light at 5 DPI.

While no substantial necrosis developed with either BeYDV or pEAQ vectors expressing GFP, leaf chlorosis appeared only with pEAQ-HT-GFP, an effect which was more pronounced at higher *Agrobacterium* concentrations (Figure 8A). As pEAQ vectors contain the 5′ and 3′ UTRs from cowpea mosaic virus, we assessed whether other viral UTRs may contribute to cell death. The 5′ UTR from tobacco mosaic virus was found to increase the cell death response compared to the native *N. benthamiana* NbPsaK 5′ UTR, despite the TMV 5′ UTR producing less recombinant protein (Figure 8B and Diamos et al., 2016). The 5′ and 3′ UTRs from pea enation mosaic virus also substantially increased cell death, while those from barley yellow dwarf virus did not (Figure 8C). These data show that certain viral untranslated regions increase the cell death response in *N. benthamiana* leaves.

## Discussion

Transient expression systems have become the most commonly used systems to produce recombinant proteins in plants (Gleba et al., 2014). While extensive work has been done to optimize the gene expression cassette and other aspects of the BeYDV system (Diamos et al., 2016; Diamos and Mason, 2018), vector replication has not been thoroughly investigated. To study replication, a modular system was created using promoters of varying strengths to express Rep and RepA at controlled levels. While the 35S promoter is widely known to drive high levels of gene expression, the NOS promoter was reported to be 30-fold weaker than the 35S in transgenic plants (Sanders et al., 1987). We found that all other promoters tested produced substantially lower Rep/RepA than 35S (Figure 1C), however, these levels were still able to provide robust accumulation of viral replicons (Figure 2) that were present in high enough quantities to be readily visible on ethidium bromide stained gels (data not shown). The potato Ubi3 promoter has been reported to have 5 to 10-fold increased activity when a reporter gene was translationally fused to ubiquitin (Garbarino and Belknap, 1994). In agreement, we found that translational fusion of Rep to ubiquitin enhanced its accumulation (Figure 1C). As geminiviruses encode few proteins, they rely heavily on host enzymes for replication. The mastrevirus wheat dwarf virus RepA preferentially forms octamers while Rep forms 6–8 subunit oligomers, which assemble at the initiation site and are thought to recruit host replication machinery (Gutierrez et al., 2004). Among the begomoviruses, tomato yellow leaf curl Sardinia virus Rep was found to form dodecamers with helicase activity (Clerot and Bernardi, 2006), and the self-interaction of Abutilon mosaic virus Rep was demonstrated *in planta* (Krenz et al., 2011). We found BeYDV Rep and RepA form high molecular weight bands consistent with the formation of oligomers comprised of 6–8 monomers (Figure 1C).

There is discrepancy in the necessity of RepA for mastreviral rolling circle replication. In cell culture experiments with wheat dwarf virus (Collin et al., 1996) or BeYDV (Liu et al., 1998; Hefferon, 2003), intron-deleted rep has been reported to support high levels of replication. In contrast, maize streak virus only supported very low levels of replication in the absence of RepA (Ruschhaupt et al., 2013). In agreement with the results of Ruschhaupt et al. (2013), we observed only low levels of replication when expressing rep alone in *N. benthamiana* leaves, even in the presence of high levels of rep (Figure 2). Despite the small increase in NVCP-expressing replicon accumulation by supplying Rep alone, a small decrease in NVCP expression was observed, perhaps indicating that replicons generated this way are less available for transcription, or that some other function of RepA increases transgene expression. Notably, expression of RepA alone also had a small negative effect on NVCP expression, indicating that both rep and RepA are indeed required for productive enhancement of transgene expression (Figure 3A). Furthermore, we also find that the relative ratio of rep and RepA is essential for replication. Expression of both rep and RepA from relatively weak promoters still resulted in robust replicon production, but this did not occur if either rep or RepA were overexpressed relative to the other (Figure 3). Rep and RepA share the same N-terminus, including DNA binding and oligomerization domains, which may permit hetero-oligomerization (Horváth et al., 1998; Missich et al., 2000). Proper hetero-oligomerization of Rep and RepA may be disrupted when either monomer is overexpressed relative to the other.

In their native configuration, production of either Rep or RepA is controlled by the excision of an intron and thus the frequency of intron removal controls the relative abundance of each protein. For maize streak virus in infected maize, it has been reported that approximately 80% of transcripts produce RepA, and only 20% produce Rep (Wright et al., 1997). We observed that 35S-driven Rep and RepA produced as much or more combined Rep/RepA than the wild-type gene (Figure 1C), yet had reduced replicon amplification (Figure 2). By reducing western blot it was possible to distinguish the 39 kDa Rep, which forms a single ∼35–40 kDa band when expressed alone, from the 33 kDa RepA, which ran as a 65–75 kDa band when expressed alone, perhaps suggestive of dimer formation (Figure 1C). We repeatedly observed that 35S-driven Rep/RepA overproduced the rep monomer-sized band and underproduced the RepA dimer-sized band compared to the wild-type configuration (Figure 1C), suggesting that 35S-driven Rep/RepA may not produce the proper ratio of each protein, thereby leading to reduced replication. However, when Rep and RepA were produced together, an additional 40–45 kDa band consistently appeared regardless of promoter (Figure 1C). As our antibody probe reacts with both Rep and RepA, it was not possible to conclusively determine the origin of this band. Interestingly, under non-reducing conditions, RepA forms two distinct high molecular weight bands when expressed alone. However, when coexpressed with Rep, only a single band was observed. This may be suggestive of different compositions of Rep and RepA present in homo-oligomers and hetero-oligomers. Further studies that can conclusively distinguish between Rep and RepA are needed to address these questions.

Plants employ the hypersensitive response as a mechanism to combat viral infection. The hypersensitive response is characterized by a burst of reactive oxygen species and the formation of necrotic lesions resulting from programmed cell death. In this study, we find that BeYDV Rep and RepA both contribute to leaf cell death, while the BeYDV MP and CP did not produce notable symptoms (Figure 4A). Furthermore, our data is suggestive of vector replication itself as a further contributor to cell death. Viral DNA sensors are well-studied components of the innate immune system in animal cells (Takeuchi and Akira, 2009), however similar sensors have not thus far been identified in plants (Zvereva and Pooggin, 2012). Alternatively, many DNA viruses have been shown to activate the DNA damage response during replication (Luftig, 2014). Here, we show that when Rep/RepA are supplied to an empty vector that has had all gene products removed but is still capable of accumulating viral replicons, the cell death response is enhanced compared to when Rep/RepA are supplied to a vector incapable of replicating (Figure 4B). However, we cannot exclude the possibility that some other cryptic component of the vector contributed to the observed cell death.

Using a controlled reduction in Rep/RepA expression, leaf cell death caused by geminiviral replicons is alleviated (Figures 3B, 5C). Despite reducing the number of available DNA templates for transcription, we found minimal reduction in the total yield of recombinant protein with non-toxic proteins (Figure 5A), and an increased accumulation of otherwise toxic proteins (Figures 3A, 5B, 6D). Several hypotheses may explain this observation. BeYDV vectors have replaced the viral movement and coat proteins with an expression cassette containing the gene of interest. During native BeYDV infection, the coat protein results in the accumulation of single-stranded viral DNA, which is packaged into virions, shuttled out of the nucleus, and, in concert with the movement protein, facilitates cell-to-cell movement and systemic spread of viral DNA (Liu et al., 2001). These interactions reduce the amount of double-stranded viral DNA available for transcription. As modified BeYDV expression vectors do not contain the movement and coat proteins, the amount of double-stranded DNA available in the nucleus to serve as a transcription template may exceed wild-type levels. Furthermore, BeYDV vectors also contain the RNA silencing suppressor P19, which likely increases the expression of Rep and RepA relative to wild-type levels. Taken together, these data suggest that more viral replicons are produced than are needed to saturate the plant transcription machinery. Therefore, we suspect reducing Rep and/or RepA expression allows a reduction in the plant hypersensitive response while still producing enough DNA templates to drive maximal transcription. By alleviating the hypersensitive response, further protein accumulation is possible for genes that otherwise would have had their production limited by cell death. Additionally, as RNA silencing and the hypersensitive response are interrelated pathways that act in concert against invading viruses, reducing the onset of hypersensitive response may also prevent premature silencing of BeYDV vectors (Zvereva and Pooggin, 2012).

The sequence context around the initiation site plays a critical role in translation (Kozak, 1999). Experiments with tobacco cells found that altering the initiation context from **C**AUAUG**C** to **A**AUAUG**G** (start codon underlined) resulted in a fourfold increase in gene expression (Ayre, 2002). While we were able to reduce cell death and increase antibody yield by reducing Rep/RepA expression, it required coinfiltration of three separate *Agrobacterium* vectors. As the native Rep gene also controls the optimum ratio of Rep/RepA by intron splicing, we reasoned that a mutation in the 5′ UTR of Rep/RepA would be a simple modification to simultaneously reduce expression of both genes while maintaining the native mechanism of controlling the relative production of Rep/RepA. The resulting vector, containing an **A**AUAUG to **C**AUAUG mutation, reduced Rep/RepA expression, reduced cell death, and provided enhanced expression of toxic proteins (Figure 6). As multiple BeYDV replicons can be placed in tandem on the same T-DNA (Huang et al., 2010), this strategy can be used to produce heteromultimeric proteins from a single vector.

*Agrobacterium* contributes to the plant cell death response in a complex manner (Hwang et al., 2015), though infiltration with higher *Agrobacterium* concentrations has often been found to contribute to cell death (Wroblewski et al., 2005). While an *Agrobacterium* OD_600_ of ∼0.2 is sufficient to deliver T-DNA to the majority of plant cells, non-replicating vector systems often use much higher concentrations of *Agrobacterium* to achieve optimum expression. This may be due to the delivery of multiple DNA copies to each cell, which serve as additional transcription templates. As replicating systems greatly amplify the input T-DNA, additional copies would be unnecessary. Sainsbury et al. (2009) reported that, when using a non-replicating vector, target protein accumulation decreased if the *Agrobacterium* concentration was reduced below an OD_600_ of 1.2, which agrees with our findings (Figure 7B). By contrast, we found no reduction in yield by reducing the *Agrobacterium* concentration from 1.2 to 0.2 using replicating BeYDV vectors (Figures 7A,B). For the expression of toxic proteins, we observed that necrosis developed when using higher *Agrobacterium* concentrations, but not with lower concentrations (Figure 7C). That this relationship was observed only with certain proteins suggests that cell death only occurs when the combined action of multiple necrosis-inducing factors reach a specific threshold. For the production of recombinant proteins with DNA-based systems, the development of cell death depends on the individual composition of the protein being produced, subcellular localization of the target protein (Howell, 2013), glycosylation of the target protein (Hamorsky et al., 2015), target protein expression level, *Agrobacterium* strain (Diamos et al., 2016) and concentration (Wroblewski et al., 2005, Figure 7D), DNA elements like matrix attachment regions (Diamos et al., 2016), 5′ and 3′ UTR elements (Figures 8B,C), viral replication elements (Figures 3B, 4B, 5C), and plant health and growth conditions (Qian et al., 2016; Matsuda et al., 2017). Modifying these factors allows enhanced accumulation of proteins that may, under less favorable conditions, elicit a cell death response. Though the mechanism by which the Rb7 MAR reduces cell death in this system is unknown, we have previously observed that larger replicons accumulate to lower amounts than smaller replicons (unpublished data), and thus incorporation of the long 1.2 kb Rb7 MAR may also reduce replicon accumulation. We anticipate these modifications will allow high-level production of other toxic biopharmaceutical proteins.

## Author Contributions

AD performed experiments and wrote the manuscript. HM helped design experiments and edited the manuscript.

## Conflict of Interest Statement

The authors declare that the research was conducted in the absence of any commercial or financial relationships that could be construed as a potential conflict of interest. The reviewer JF and handling Editor declared their shared affiliation.

## Supplementary Material

Figure S1 The full Rep/RepA western blot from which part of Figure 1 was derived. S1, S2, and S3 indicate different leaf samples.

Figure S2 The full Rep/RepA western blot from which part of Figure 1 was derived. TMV, a tobacco mosaic virus vector expressing either Rep or RepA.
