# Supplementary figures and images for: Modifying the Replication of Geminiviral Vectors Reduces Cell Death and Enhances Expression of Biopharmaceutical Proteins in Nicotiana benthamiana Leaves
Source: Front Plant Sci. 2019 Jan 9;9:1974. doi: 10.3389/fpls.2018.01974 (PMC6333858; doi:10.3389/fpls.2018.01974)

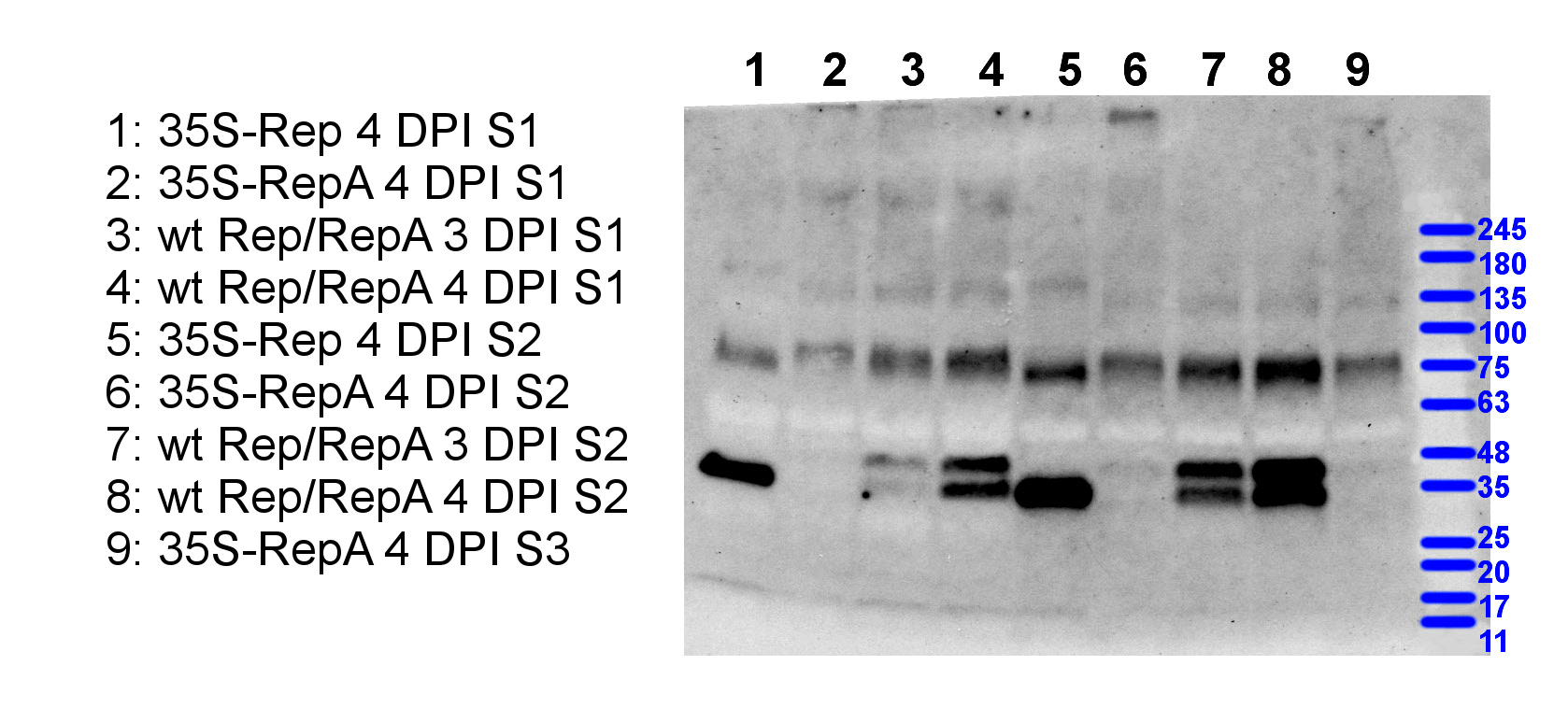

Supplement: Figure S1 — The full Rep/RepA western blot from which part of Figure 1 was derived. S1, S2, and S3 indicate different leaf samples. [file Image_1.jpg]

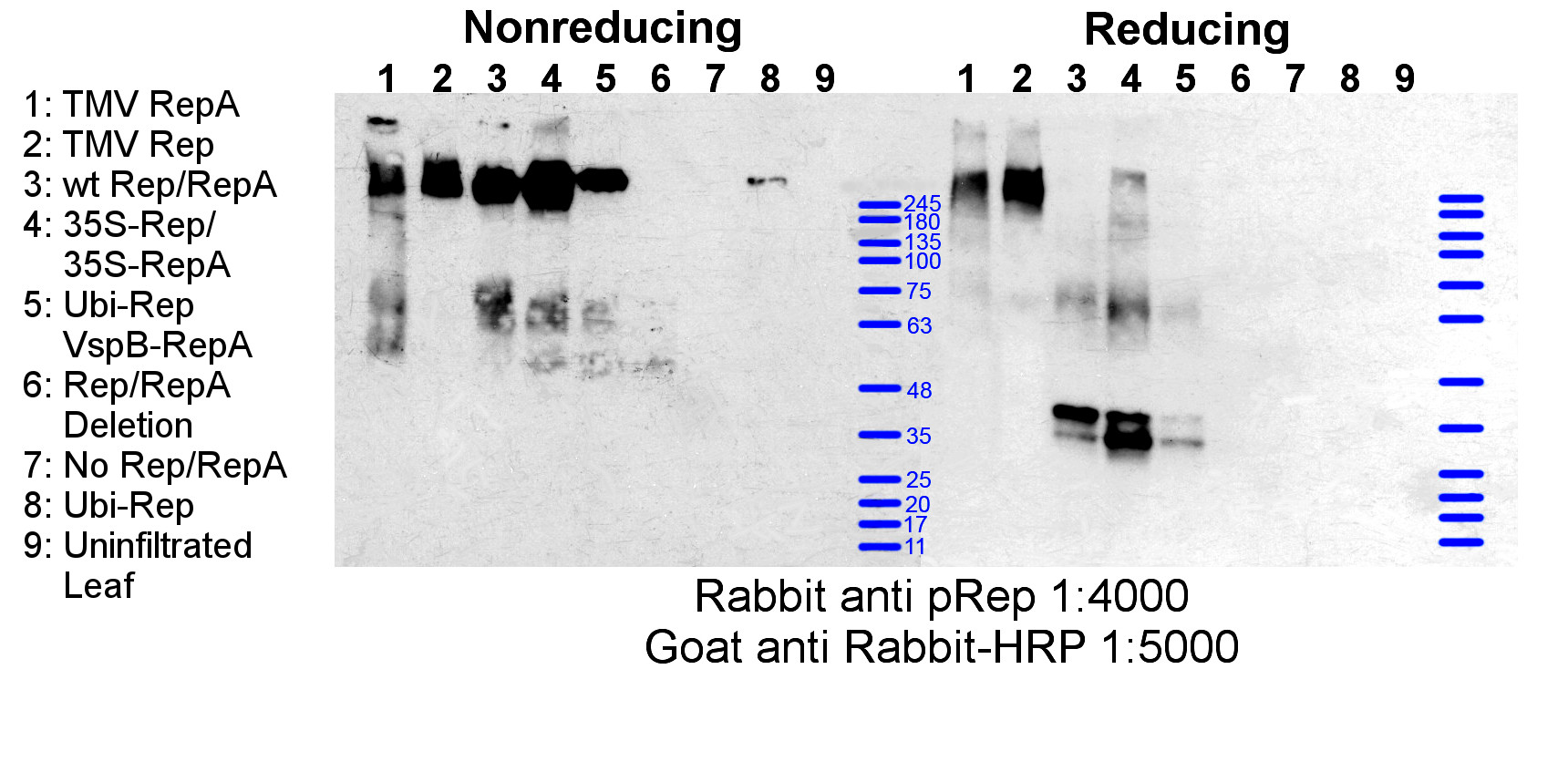

Supplement: Figure S2 — The full Rep/RepA western blot from which part of Figure 1 was derived. TMV, a tobacco mosaic virus vector expressing either Rep or RepA. [file Image_2.jpg]
